# Plant Responses Underlying Timely Specialized Metabolites Induction of *Brassica* Crops

**DOI:** 10.3389/fpls.2021.807710

**Published:** 2022-02-03

**Authors:** Maroua Doghri, Víctor Manuel Rodríguez, Daniel J. Kliebenstein, Marta Francisco

**Affiliations:** ^1^Misión Biológica de Galicia (MBG-CSIC), Pontevedra, Spain; ^2^Department of Plant Biology, Faculty of Biology, Institute of Biotechnology and Biomedicine, University of Valencia, Valencia, Spain; ^3^Department of Plant Sciences, University of California, Davis, Davis, CA, United States; ^4^DynaMo Center of Excellence, University of Copenhagen, Frederiksberg, Denmark

**Keywords:** *Brassicaceae*, timely induced resistance, herbivory, *Mamestra brassicae*, wounding, metabolomics

## Abstract

A large subset of plant stress-signaling pathways, including those related with chemical defense production, exhibit diurnal or circadian oscillations. However the extent to which diurnal or circadian time influences the stress mediated accumulation of plant specialized metabolites remains largely unknown. Because plant responses to physical stress (e.g., wounding) is considered a common component of mounting a response against a broad range of environmental stresses, including herbivory, we have utilized mechanical wounding as the stress stimulus to determine the direct contribution of time of day on the induced defenses of *Brassica* crops. We analyzed glucosinolates (GSLs) from leaves of broccoli (*Brassica oleracea*) and turnip greens (*Brassica rapa*) following exposure to mechanical wounding at dawn (ZT0), mid-day (ZT4), and dusk (ZT8). Several GSLs differentially accumulated and their changes depended upon the time of day at wounding was performed. This response varied considerably between species. In a parallel experiment, we investigated whether diurnal activation of *Brassica* phytochemicals in response to wounding might prime plants against herbivore attack. Results showed that maximal response of plant chemical defense against larvae of the generalist pest *Mamestra brassicae* occurred at ZT0 in broccoli and ZT8 in turnip greens. Metabolome analysis for global trends of time dependent compounds showed that sulfur-containing phytochemicals, GSL hydrolysis products, auxin-signaling components, and other metabolites activators of plant disease resistance (nicotinamide and pipecolate) had important contributions to the responses of *M. brassicae* feeding behavior in broccoli at morning. Overall, the findings in this study highlight a significant role for time of day in the wound stress responsive metabolome, which can in turn affect plant-herbivore interactions.

## Introduction

Plants are constantly constrained by a wide array of biotic and abiotic stresses, of which damage caused by chewing insects greatly affects plant performance and survival. To defend themselves, plants have evolved a sophisticated defensive system allowing them to perceive mechanical injury and trigger an induced defensive response. Wounding in the plant tissue and/or herbivore feeding cues invokes a reprogramming of defense-related gene expression including genes required for *de novo* biosynthesis of herbivore-induced chemical defenses (Savatin et al., 2014; Jacobo-Velázquez et al., 2015; Body et al., 2019; Guan et al., 2021). An example of this are the glucosinolates (GSLs), a specific class of secondary compounds present in plants of the *Brassicaceae* family that provide resistance to generalist herbivores. This defensive system imposes an energetic and metabolic cost to the plant since energy and resources are diverting away from other plant processes such as growth or primary metabolism toward generating these specialized compounds (Agrawal et al., 2002; Paul-Victor et al., 2010). Thus, it is crucial for the plant to sacrifice this investment only when it is necessary.

One mechanism that dynamically adjusts plant overall metabolism in anticipation of a highly predictable environmental cues is the circadian clock. The circadian clock is an endogenous timekeeper that orchestrates rhythmic behaviors of physiological, metabolic and developmental processes in consonance with external cyclic events (McClung, 2019). In plants, the circadian clock is crucial to cope with daily fluctuations of light intensity, temperature and humidity. In addition, herbivores, pathogens and pollinators rely on endogenous clocks and therefore represent predictable biotic threats. Several lines of evidence indicate that large subsets of biotic and abiotic defense-signaling pathways in plants are clock regulated. Genes from defense pathways show rhythmic expression and their transcriptional response often depends on the time of the day at which the plants are exposed to the stress (Bhardwaj et al., 2011; Wang et al., 2011; Zhang et al., 2013). For instance, Walley et al. (2007) showed that wound responsive genes, inducing both biotic and abiotic stress responses, are circadian regulated with consolidated phases of peak expression at certain times of the day. In concordance, the production of wound-induced phytohormones such as Jasmonic acid (JA) and Salicylic acid (SA), as well as downstream activation of plant defense metabolites, including GSLs, is also dependent on circadian periodicity (Covington et al., 2008; Goodspeed et al., 2012, 2013; Soengas et al., 2018a,b; Lei et al., 2019).

It has been hypothesized that plants utilize the clock as a strategy to anticipate the timing of potential threats to phase basal defenses to the particular time of the day when they are most likely to be needed (Harmer et al., 2000; Goodspeed et al., 2012; Burow and Halkier, 2017). However, to date there is only a handful of studies addressing the circadian connection between plant defense signaling pathways and the timing of pest attack. Goodspeed et al. (2012), reported that Arabidopsis resistance against the generalist herbivore insect *Trichoplusia ni*, is highly dependent on clock-regulated diurnal accumulation of endogenous JA. In a complementary experiment, they demonstrated that cyclic activation of GSLs is required for this phase-dependent resistance (Goodspeed et al., 2013). Consistently, Lei et al. (2019) and Lei and Zhu-Salzman (2021) reported that the link between the circadian clock and plant defense against green peach aphids *Myzus persicae* is mediated by the cyclic expression pattern of key indole GSL biosynthetic genes. It is well known that wounding or herbivory often induces JA levels and in consequence GSLs compared with their basal levels (van Dam et al., 2004; Jansen et al., 2009). However, to what extent the dimension of time and/or the circadian clock contribute to the synthesis and mobilization of plant induced resistant compounds under stress responses remains largely unknown.

The aim of this work is to begin providing more insight into how temporal dynamics influence the synthesis and mobilization of induced chemical defenses and if temporal variability in defensive response may alter future rates of herbivory. To start to solve these questions, this work studied how wounding stress is influenced by crosstalk with the diurnal cycle in two different crop species of the *Brassicaceae* family, broccoli (*B. oleracea*) and turnip greens (*B. rapa*). In particular, we first examined plant defense capacity (measured in terms of GSL content) in response to wounding stress, and included “time of day” as a controlled factor. Our work revealed that induction of plant defensive compounds in response to a repetitive damage exerted by wounding depends on the time of the day at which wounding takes place and this response differed between species. We further demonstrated that timely activation of *Brassica* crops phytochemicals by wounding might prime plants against future herbivore attack. Global metabolome analysis of leaves harvested at the time of maximal resistance against the generalist pest *Mamestra brassicae* indicated that timely induced sulfur-containing phytochemicals, GSLs breakdown products, auxin-signaling components, and other metabolites activators of plant resistance such as nicotinamide and pipecolate are behind this response. Together, these data highlight a significant role for time of day in the induced plant defensive compounds affecting plant-herbivore interactions.

## Materials and Methods

### Plant Growth and Harvest Conditions

**FIGURE 1:**
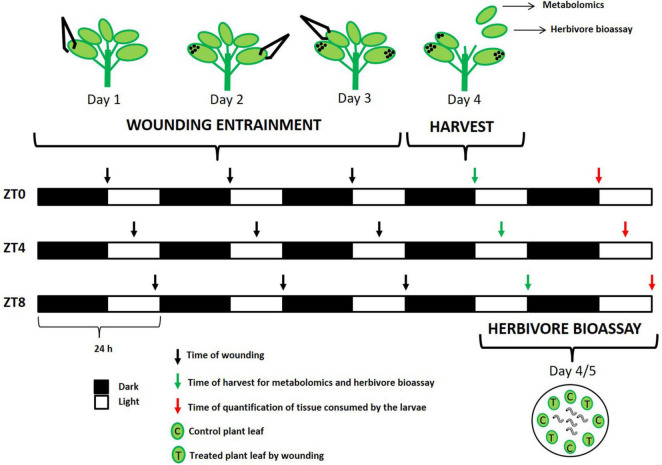
Scheme of the wounding treatment at different time points (ZT0, dawn; ZT0, mid-day; ZT8, dusk). Plants were wounded during three consecutive days and harvested at fourth day. Black arrows represent the time of day at which wounding was performed. Green arrows represent the time of day at which leaves were harvested for metabolomics and herbivore bioassay. Red arrow represents the time of day at which quantification of tissue consumed by the larvae was done for each experiment. Black dots on the leaves indicate previously wounding leaves.

Seeds of a commercial hybrid of broccoli “green calabrese” (*B. oleracea* var. *botrytis*) (Batlle S.A., Barcelona) and a local variety of turnip greens “globo blanco de Lugo” (*B. rapa* ssp. *rapa*) (WAM S.L.L, Pontevedra) were planted in potting soil stratified at 4°C in the dark for 3 days to optimize germination. Plants were sown in a randomized block design consisting of 72 pots (4 × 4 cm) per genotype, each containing one plant. All plants were grown in short-day conditions (8/16 h light/dark) in a growth chamber under controlled conditions (20°C of temperature and 80% of humidity). Because it has been hypothesized that plant responses to physical stress is a mechanism common to mounting a response against a broad range of environmental stresses, including herbivory (Walley et al., 2007), we utilized mechanical wounding as the stress stimulus. To study how time of day effects plant induced resistance in response to wounding, sampling and treatments were performed at various Zeitgeber times (ZT) (Figure 1). At the five-leaf stage, each pool of plants [12 plants × treatment (wounded and control) × time-point × genotype] were exposed to mechanical wounding simulating three different day-time attacks: dawn (ZT0), mid-day (ZT4), and dusk (ZT8) during three consecutive days (indicated in Figure 1). For wounding treatments, we used laboratory forceps damaging the third superior part of the leaf, which effectively wounded ∼30% of the leaf area. One leaf per day was wounded starting from the oldest. For the control treatment, plants were kept unwounded. On the fourth day, at the same time-point that each pool of plants was trained to receive damage (ZT0, ZT4, and ZT8) the two youngest leaves per plant were harvested from each genotype and treatment. One leaf was used for metabolite analysis and the other one was used for feeding experiments. For metabolite analysis, individual leaves were cut, placed in plastic scintillation vials and immediately frozen in liquid nitrogen and stored at −80°C. Frozen samples were lyophilized and ground to a fine powder using an electric mill and stored in tubes until extraction and analysis.

### Insects

*Mamestra brassicae* eggs were supplied by the Centre de Recherche de Versailles (Institut National de la Recherche Agronomique, France) and incubated at 20°C until they hatched. Neonate larvae were maintained on cabbage leaves under laboratory conditions until the bioassays were set up. To test whether the larvae feeding activity is affected by the light/dark cycle, eight petri dishes containing three leaf-disks of broccoli and turnip greens (separately) were placed with three *M. brassicae* larvae. Larvae were entrained to 12 h light 12 h dark cycles for 2 days prior to testing. The remaining leaf area after insect feeding was recorded at the end of the light period and the end of the dark period during two consecutive days. The leaf area in each experiment was measured using the software ImageJ (Schneider et al., 2012). We found that *M. brassicae* larvae feed similarly during light and dark period under constant temperature conditions (data not shown).

### Herbivore Bioassay

For feeding experiments, three disks of 2 cm diameter from control and treated leaves were placed in alternate order in a Petri dish along with five two-instar larvae of *M. brassicae* L. (Noctuidae) (Figure 1). We used four replicates for each treatment per genotype at each time-point. Petri dishes were placed in the growth chamber under the same environmental conditions used for plant growth. Larvae were allowed to eat during 24 h. We recorded pictures of each disk using a digital camera (Nikon COOLPIX P100) to quantify the amount of tissue consumed by the larvae. Leaf area was measured using the software ImageJ (Schneider et al., 2012). Data were analyzed using analyses of variance (ANOVA) and mean comparisons were performed through the Least Significant Difference (LSD) test (*p* < 0.05) conducted with the GLM procedure using the SAS software (SAS Institute, Cary, NC, United States).

### Glucosinolates Extraction and Quantification

Sample extraction and desulfation, were performed according to Kliebenstein et al. (2001) with minor modifications. Ten microliters of the desulfo-GSL extract were used to identify and quantify the GSLs. The chromatographic analyses were carried out on an Ultra-High-Performance Liquid-Chromatograph (UHPLC Nexera LC-30AD; Shimadzu Corporation, Kyoto, Japan) equipped with a Nexera SIL-30AC injector and one SPD-M20A UV/VIS photodiode array detector. The UHPLC column was XSelect HSS T3 XP ColumnC18 protected with a C18 guard cartridge. The oven temperature was set at 30°C. Compounds were separated using the following method in aqueous acetonitrile, with a flow of 0.5 mL min^–1^: 1.5 min at 100% H_2_O, an 11 min gradient from 0 to 25% (v/v) acetonitrile, 1.5 min at 25% (v/v) acetonitrile, a minute gradient from 25 to 0% (v/v) acetonitrile, and a final 3 min at 100% H_2_O. Data was recorded on a computer with the LabSolutions software (Shimadzu, Corporation, Kyoto, Japan). All GSLs were quantified at 229 nm by using glucotropaeolin (GTP, monohydrate from Phytoplan Diehm & Neuberger GmbH, Heidelberg, Germany) as internal standard and quantified by comparison to purified standards. We reported the concentration (nmol per g of DW) of individual GSL compounds as well as the sums of total aliphatic and indolic GSLs, two classes of GSLs based on the amino acid from which they have derived. ANOVA was used to compare GSL traits variation between control and treated plants by the GLM procedure using the SAS software (SAS Institute, Cary, NC, United States).

### Untargeted Metabolomics Analysis

Extraction was performed using 20 mg of lyophilized powder with 1 ml of 80% aqueous methanol and vortexed for 10 s for homogenization. Extracts were sonicated in an ultrasonic bath (3510E-MTH, Bransonic^®^, Mexico) for 5 min at 30 Hz frequency and then centrifuged for 10 min at maximum speed (20,000 × *g*) at room temperature. The supernatant was transferred to a clean tube. To extract the maximum of metabolites, a second extraction was performed, starting with the pellet and operating similarly as mentioned above, 200 μl from the resulting supernatant were filtered using a 0.22-micrometer micropore PTTE filter and placed in HPLC vials containing 0.8 ml of 80% aqueous methanol. Five microliters of each sample were injected into an ultra-high-performance liquid chromatography (UHPLC) system (Thermo Dionex Ultimate 3000 LC) connected to a QTOF detector (Bruker Compact™) with a heated electrospray ionization (ESI) source. Chromatographic separation was performed in a Bruker UHPLC Intensity Solo 2 C18 2.1 × 100 mm 1.7 μm pore size column using a binary gradient solvent mode, consisting of 0.1% formic acid in water (solvent A) and acetonitrile (solvent B). The following gradient was used: 3% B (0–3 min), from 3 to 25% B (3–10 min), from 25 to 80% B (10–18 min), from 80 to 100% B (18–22 min), and held at 100% B until 24 min. The flow rate was established at 0.3 mL min-1 and the column temperature was controlled at 35°C. MS data were acquired using an acquisition rate of 2 Hz over the mass range of 50–1,200 m/z. Both polarities (±) of ESI mode were used under the following specific conditions: gas flow 8 L min^–1^; nebulizer pressure 38 psi; dry gas 9 L min^–1^; dry temperature 220°C. Capillary and end plate offset were set to 4,500 and 500 V, respectively. LC-qTOF system stability was tested by three consecutive injections of chloramphenicol (ESI – mode; ΔRT = 0.01 min; Δm/z = 0.002) and triphenyl phosphate (ESI + mode; ΔRT = 0.02 min; Δm/z = 0.001). MS/MS analysis was performed in pooled samples (per day and condition). This analysis was operated in a spectrum acquisition range from 50 to 1,200 m/z using an acquisition rate of 8 Hz. Ions were targeted based on the previously determined accurate mass and retention time (RT) and fragmented by using different collision energy ramps to cover a range from 15 to 50 eV. Raw data from the UHPLC-QTOF were uploaded and processed by the commercial software MetaboScape 4.0 from Bruker (Kessler et al., 2016). Peak detection and data alignment were proceeded automatically using the T-ReX 3D algorithm. The resulted features matrix was imported to MetaboAnalyst (Chong et al., 2019). Data were normalized through Pareto scaling to avoid unwanted systemic biases (reducing the masking effect from abundant metabolites) with conserving data structure, keeping it partially intact (van den Berg et al., 2006; Yang et al., 2015). We performed two statistical analysis (multivariate and univariate analysis) to identify the most relevant features in the separation between the wound and control group of plants. These two methodologies may provide complementary biomarkers that could help to give a general overview of metabolic changes occurring in the plants (Saccenti et al., 2014). Multivariate analysis was performed using supervised partial least squares discriminate analysis (PLS-DA) methodology. Selection of more discriminant features was performed using a threshold of VIP (variable importance in projection) score >2. Univariate analysis was performed using a Student *t*-test. To control the proportion of false positives among significant results that can be obtained due to multiple hypothesis testing we used false discovery rate (FDR ≤ 0.05). Features with an FDR ≤ 0.05 and a | log2FC (fold-change)| ≥ 1 were considered differentially regulated. Metabolite identification was performed based on the exact mass and MS/MS spectrum. The software MetaboScape 4.0 (Bruker Daltonics, Germany) and SIRIUS 4 (Dührkop et al., 2019) were used to elucidate the metabolite molecular formula with default parameters. Both software use the exact mass and isotopic pattern to calculate the most probable molecular formula for each compound. In this analysis, only C, H, O, N, S, and P were allowed to determine the molecular formula. Formulas with a mass deviation <5 ppm and a mSigma <20 (MetaboScape) were considered for annotation. For a tentative identification, publicly available metabolites databases were used [PubChem (Kim et al., 2021), KEGG (Kanehisa et al., 2021), CheBi (Hastings et al., 2016), ChemSpider (Pence and Williams, 2010), Metlin (Guijas et al., 2018)].

## Results

### Time of Day Contributes to Differential Phytochemical Responses to Mechanical Wounding

**FIGURE 2:**
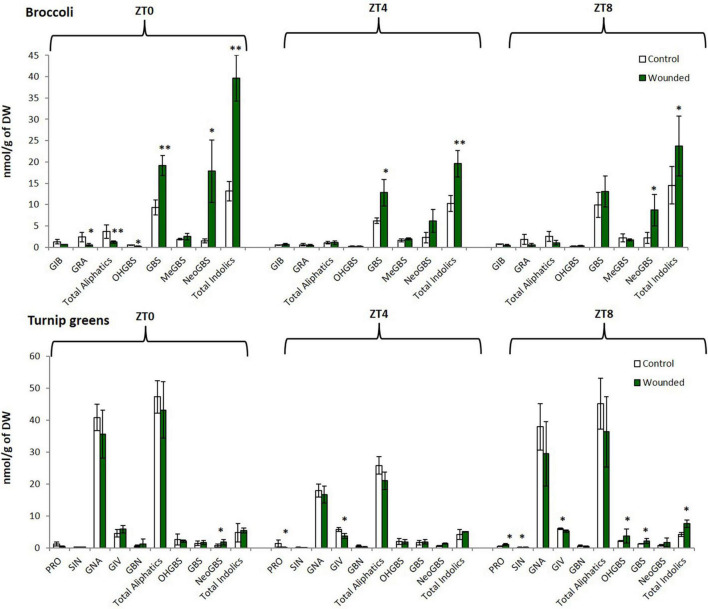
Glucosinolate content in a commercial hybrid of broccoli “green calabrese” (*B. oleracea* var. *botrytis*) and a local variety of turnip greens “globo blanco de Lugo” (*B. rapa* ssp. *rapa*) at different time points (ZT0, dawn; ZT0, mid-day; ZT8, dusk). Error bars represent ± standard error (SE). GIB, glucoiberin; GRA, glucoraphanin; PRO, progoitrin; SIN, sinigrin; GNA, gluconasturtiin; GIV, glucoiberverin; GBN, glucobrassicanapin; GBS, glucobrassicin; OHGBS, hydroxyglucobrassicin; MeGBS, methoxyglucobrassicin; NeoGBS, neoglucobrassicin. The asterisk indicates statistically significant differences between mean values according to Student’s *t*-test (^∗^*p* < 0.05; ^∗∗^*p* < 0.001).

We found significant variation among control and treated *Brassica* plants for individual and total GSLs (Figure 2). Our work revealed that changes on these defense-related compounds levels was dependent upon the time of day at which wounding was performed, and this response varied among species. Specifically, for broccoli the greatest changes on GSLs levels were found when wounding occurred at ZT0 (Figure 2). At this time point total and individual indolic GSLs were highly induced, for instance the content of Neoglucobrassicin (N-methoxy-3-indolyl glucosinolate) from wounded plants was about 12-fold higher than control. In contrast, aliphatic GSLs significantly decreased in broccoli (*B. oleracea*) plants wounded at ZT0 while aliphatic GSLs did not change when wounding occurred at the other time-points. For turnip greens (*B. rapa*), most of the significant differences in GSLs traits were found when wounding occurred at ZT8 and the trend were similar to that found for broccoli, indole GSLs increased after wounding and aliphatic GSLs tend to decrease (Figure 2). These results indicate that the wound activation of distinct sets of defense metabolites is clock influenced in *Brassica*.

### Differential Plant Defense Response to the Time of Wounding Can Alter *Mamestra brassicae* Feeding Behavior

**FIGURE 3:**
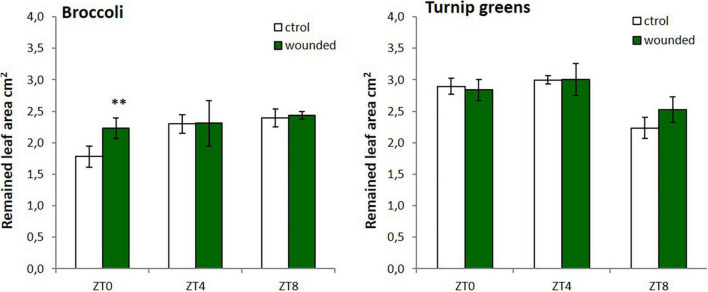
Average area of leaves disk remaining after 24 h of *Mamestra brassicae* L. feeding. Error bars represent ± standard error (SE). Leaves were collected from plants wounded at dawn (ZT0), mid-day (ZT4), or dusk (ZT8) during three consecutive days. The asterisk indicates statistically significant differences between mean values according to Student’s *t*-test (^∗∗^*p* < 0.001).

To test whether the time-dependent accumulation of GSLs after wounding could also prime plants against insect feeding we performed a bioassay. In spite of different accumulation of GSLs in response to wounding, we only observed significant differences on larvae intake (measured as remained leaf area) among control and treated broccoli leaves at ZT0 (*P*-value = 0.0079) (Figure 3). For turnip greens, differences for larval food intake were found at ZT8, although it was not statistically significant (*P*-value = 0.053). This trend was not observed at other time points (Figure 3). Thus, maximal responsiveness of plant defense against *M. brassicae* is stimulated by wounding at ZT0 in broccoli and at ZT8 in turnip greens. These results indicate that the effect of the time-dependent wounding on herbivory can only partly be mediated by GSLs and that other factors must be involved in the response.

### Identifying Plant Metabolites Involved in *Brassica* Defense Using an Untargeted Metabolomics Approach

To define a comprehensive landscape of metabolite reprogramming underlying time-of-day induced resistance of *Brassica* crops, we carried out an untargeted metabolomics analysis using UPLC-Q-TOF-MS/MS. We analyzed global trends of metabolite variations by searching for changes that occur in leaves from ZT0 and ZT8 wound-entrained plants compared with control plants for broccoli and turnip greens, respectively. We focused on ZT0 and ZT8 because they were the samples with larval food intake differences. A total of 466 features in broccoli leaves and 619 features in turnip greens leaves were detected. The PLS-DA score plot shows no discrimination between samples for turnip greens, indicating that wounding entrainment at ZT8 had no visible effect on the global metabolite profiles of wounded plants. We did, however, find a discernible metabolite separation between control and unwounded plants at ZT0 in the broccoli leaves. Therefore, further analysis and metabolite annotation are referred to broccoli samples.

**FIGURE 4:**
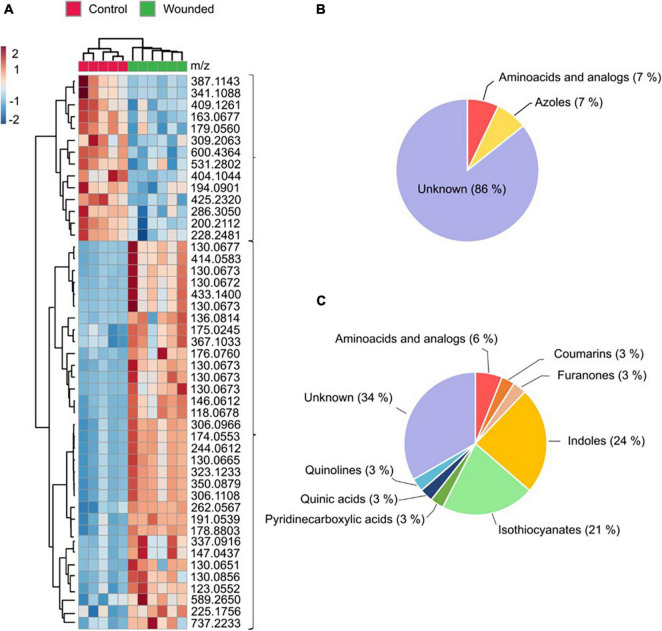
Identification of metabolites involved in the wounding defensive response in broccoli (*B. oleracea* var. *botrytis*). Samples were harvested at dawn (ZT0). **(A)** Cluster heatmap analysis of the intensity of 47 metabolites differentially accumulated in response to wounding. **(B)** Grouping of cluster of metabolites with lower accumulation in wounded plants based on the ClassyFire chemical taxonomy. **(C)** Grouping of cluster of metabolites with higher accumulation in wounded plants based on the ClassyFire chemical taxonomy. The scale indicates the color code relative to the normalized metabolite abundance (ranging from –2 up to 2).

Among the 466 ions significantly altered in broccoli, 47 were selected as important features (22 based on VIP-score values of the PLS-DA analysis and 25 based on the volcano-plot analysis) contributing to the ZT0 wound response (Supplementary Table 1). A hierarchical cluster analysis grouped these 47 metabolites into two separate groups (Figure 4A). One group encompasses metabolites with a lower accumulation in unwounded leaves of plants wounded at ZT0 compared to that of the control plants. We were unable to assign a candidate compound to most of these compounds (Figure 4B). The other group encompasses metabolites with higher accumulation in unwounded leaves of plants wounded at ZT0. Most of these compounds are classified as indoles or indole derivates or isothiocyanates (Figure 4C) based on the ClassyFire chemical taxonomy (Djoumbou Feunang et al., 2016).

## Discussion

Circadian clock regulation of stress signaling allows the plant to prepare for unpleasant events and to gate suitable responses in a time-of-day manner. Our results show a direct contribution of time of day on the ability of wounding to systemically induce specialized metabolism of *Brassica* crops. Within a fixed light/dark cycle, plant defense response to wounding elicitation depended on the diurnal timing of the wounding (Figure 2). Induction of indole GSLs at dawn for broccoli and dusk for turnip greens was 2–12 times higher when compared with wounding during the day. Remarkable, the time-of-day response of GSLs varied by genotype. Previous work has also shown that wound or other herbivory responses vary across species and genotypes in their circadian or diurnal response. Arimura et al. (2008) showed that nocturnal damage to lima bean leaves increased JA levels 2–3 times higher when compared with day damage; In contrast, sensitivity to JA in Arabidopsis plants is highest at dawn (Baldwin and Meldau, 2013). In *Nicotiana attenuata*, the peak accumulation of several herbivory-induced defense metabolites, after simulated herbivory varies between local and systemic tissues, leaves and roots (Kim et al., 2011). Natural variation on diurnal and/or circadian regulation of plant defense is likely to have ecological and breeding significance because plants may have been selected to maximize its anti-herbivore defenses to the time-of-day that is most valuable or vulnerable to attack. Therefore, we hypothesized the existence of interconnections among temporal induction of specialized metabolites and herbivore performance.

The Lepidopteran insect *M. brassicae* is a generalist insect that feeds on plants from several species belonging to more than 20 families, of which members of the *Brassicaceae* are among the most preferred (Rojas et al., 2000; Cartea et al., 2009). Feeding by the caterpillars causes severe damage to the plants, and it is an economically devastating pest in agriculture (Cartea et al., 2010). Although it is documented that the moth of this insect is most active at night (Devetak et al., 2010), we found that at the larvae stage, under constant temperature conditions they feed similarly during light and dark periods. Hence, any change in the feeding preference of larva under the same conditions could be attributed to the differential chemical composition of the plant. Earlier reports have been shown that *M. brassicae* preference and performance are both affected by GSLs content and composition when comparing genotypes in choice and non-choice feeding experiments (Cartea et al., 2010; Jeschke et al., 2017; Badenes-Pérez and Cartea, 2021). However, how wounding and time-of-day may prime the plant to influence herbivore performance has not been previously reported. Here we demonstrated that the time of wounding alters *M. brassicae* feeding behavior in time and genotype-dependent manner. *M. brassicae* caterpillars consumed less leaf material when broccoli was wounded at ZT0 while in turnip greens the lowest consumption was found in plants wounded at ZT8. This correlates with the treatment that maximized GSL induction accumulation in each crop (Figures 2, 3). This suggests that plants may be adapted to use the circadian clock to schedule and gate defense to match the likely timing of an attack under natural conditions.

Further dissection of the metabolic reconfiguration shed some light on the time-of-day influence over wound induced defense network of broccoli plants against *M. brassicae.* This analysis showed that GSLs breakdown products, IAA components, NAD intermediates and pipecolic acid were the identified metabolites that most significantly contributed for the chemical induced response of broccoli at dawn (Figure 4 and Supplementary Table 1). In turnip greens, there was no clear effect of wounding on the global metabolite profiles.

**FIGURE 5:**
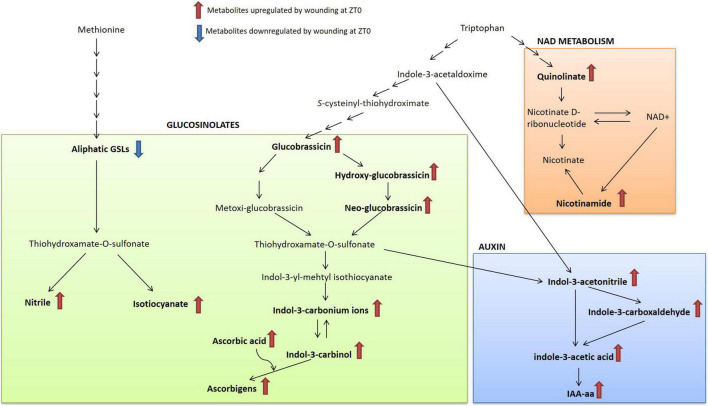
Schematic representation of the major metabolic pathways activated in response to wounding in broccoli (*B. oleracea* var. *botrytis*). Metabolites in bold show a significant variation after wounding treatment. NAD, nicotinamide adenine dinucleotide; IAA-aa, Indole-3-acetic acid conjugated by amide linkage to amino acids.

Upon tissue damage, such as herbivory or wounding, GSL come into contact with myrosinase enzyme (thioglucoside glucohydrolase EC 3.2.1.147). The hydrolysis originates an unstable intermediary (thiohydroxamate-*O*-sulfonate) that decomposes into a series of products such as ITCs, nitriles, thiocyanates, epithionitriles and oxazolidines depending on the chemical nature of the parent GSL, the presence of other different enzymes, the pH and cofactors (Wittstock and Halkier, 2002). Metabolomics analysis reveals that ITCs, nitriles and other downstream bioactive compounds were up-regulated in broccoli wounded leaves at morning (Figures 4, 5). The ITCs are highly reactive and can react with water, ascorbate, glutathione, amino acids, and other plant metabolites to produce a variety of physiologically active compounds associated with herbivore and pathogen defense (i.e., indole-3-carbinol) (Clay et al., 2009). On the contrary, the nitrile formation pathway has been proposed as a possible route of non-toxic GSLs turnover to rebuild primary metabolites (Kissen and Bones, 2009), although recently they seem to be involved into a coordinated crosstalk with phytohormone signaling (Katz et al., 2020; Ting et al., 2020). Over all, our results indicated that the plant may be more prone to trigger a GSL-related defense response when wounding occurs during the morning. Supporting this notion, Brandt et al. (2018) observed a diurnal rhythm for myrosinase activity with a significant increase of activity at the beginning of the light period in Arabidopsis. Further research is necessary to dissect the direct connections between GSL catabolism and the clock and test whether this response can be extended to other *Brassica* crop species.

Interestingly, the growth regulator IAA, a natural auxin, has been shown to be up-regulated in response to wounding (Figure 5). IAA is necessary for the proper development of embryos, roots, and shoots, and is also well known for its role in gravitropism and phototropism (Ludwig-Müller, 2011). Early studies reported an apparent antagonism between IAA and plant immunity against wounding and/or herbivory (Erb et al., 2012; Casalongué et al., 2014; Machado et al., 2016). This is explained for the involvement of auxins in the trade-off between growth and defense (Naseem et al., 2015). However, here we demonstrated that broccoli plants entrained to receive an attack during the morning increased their IAA levels altogether with specialized defensive compounds. This indicates that plants may have different strategies depending on the time of day at which damage occurs. This potential is supported by recent research suggesting that IAA accumulation is a rapid and specific signal that regulates a subset of systemic, hormone-dependent secondary metabolites under herbivore-attacked plants (Machado et al., 2016). Supporting this hypothesis, we also detected a possible relationship between GSL catabolism and IAA homeostasis in response to wounding.

One possible connection between GSL catabolism and IAA homeostasis is that indolic GSL can catabolize to indole-3-acetonitrile and then further converted into auxin, and all the intermediary metabolites of this pathway were up-regulated under wounding stress at dawn in broccoli (Figure 5). A tendency of IAA accumulation in accordance with turnover of indolic GSLs has been previously observed (Müller and Weiler, 2000; Reintanz et al., 2001). It has been suggested that this pathway is likely to be operative only under special environmental circumstances (Malka and Cheng, 2017). However, the physiological role of this interaction is so far not conclusive. The fact that promotion of auxin accumulation in coordination with GSL breakdown product reduced the herbivore preference of wounded leaves, strongly suggest that GSL-auxin homeostasis is an important determinant of broccoli tolerance against herbivory. A deeper knowledge of this response would allow optimizing cultural practices against pests to optimize plant fitness in the field.

A closer examination of untargeted metabolomic analyses revealed that other metabolites, such as nicotinamide and pipecolate, critical regulators of inducible plant immunity (Návarová et al., 2013; Miwa et al., 2017; Sidiq et al., 2021) as well as the naturally occurring sulfur compound, SMCSO, were also involved on the broccoli defense priming (Figure 5). Even though these metabolites appear to act as modulators in the coordination of defense responses against biotrophic and necrotrophic pathogens, here we report that they may serve as novel priming agent for insect pest control in crop production. Results from this work will facilitate future research investigating how specialized metabolites respond to a diel-dependent repetitive stress.

As a result of millions of years of co-evolution plants have evolved sophisticated mechanism to anticipate and protect themselves against insect attack. Our results indicate that the daily rhythmicity of insect feeding allow the plant to accumulate defensive compounds prior to the highest peaks of insect activity. The fine-tuning of this response is reflected in the differential response observed among species. At the molecular level, the preventive response involved mainly accumulation of GSLs and related compounds along with indoles involved in the biosynthesis of auxins.

## Data Availability Statement

The original contributions presented in the study are included in the article/Supplementary Material, further inquiries can be directed to the corresponding author/s.

## Author Contributions

MF and VR conceived and designed the experiments. MD, MF, and VR assisted with the plant trials setup, conducted the laboratory work, and did the metabolomics analysis. MD, MF, DK, and VR analyzed the data and wrote the manuscript. All authors read and approved the final manuscript.

## Conflict of Interest

The authors declare that the research was conducted in the absence of any commercial or financial relationships that could be construed as a potential conflict of interest.

## Publisher’s Note

All claims expressed in this article are solely those of the authors and do not necessarily represent those of their affiliated organizations, or those of the publisher, the editors and the reviewers. Any product that may be evaluated in this article, or claim that may be made by its manufacturer, is not guaranteed or endorsed by the publisher.

## Supplementary Material

Supplementary Figure 1 **(A)** Multivariate analysis using supervised partial least squares discriminate analysis (PLS-DA) in separation of control and wounded leaves from broccoli leaves harvested at ZT0. **(B)** Cross validation analysis indicated that two components model was the optimal model for broccoli samples. **(C)** PLS-DA analysis of control and wounded leaves from turnip greens harvested at ZT8. **(D)** Cross validation analysis indicated no discrimination between turnip greens samples. Colored circles represent 95% confidence intervals. Colored dots represent individual samples. The component that best classifies the model is shown with red asterisk.

Supplementary Figure 2 Volcano plot displaying features that were most differentially expressed at wounded broccoli plants relative to control at ZT0.

Supplementary Table 1 Metabolomic dataset of features detected in broccoli leaves using UPLC-Q-TOF-MS/MS.

Supplementary Table 2 Metabolomic dataset of features detected in turnip greens leaves using UPLC-Q-TOF-MS/MS.

Supplementary Table 3 Metabolomic dataset of important features (based on VIP-score values of the PLS-DA analysis and on the volcano-plot analysis) contributing to the responses of wounding and *M. brassicae* feeding behavior from broccoli samples at ZT0.
