# Supplementary figures and images for: Plant Responses Underlying Timely Specialized Metabolites Induction of Brassica Crops
Source: Front Plant Sci. 2022 Feb 3;12:807710. doi: 10.3389/fpls.2021.807710 (PMC8850993; doi:10.3389/fpls.2021.807710)

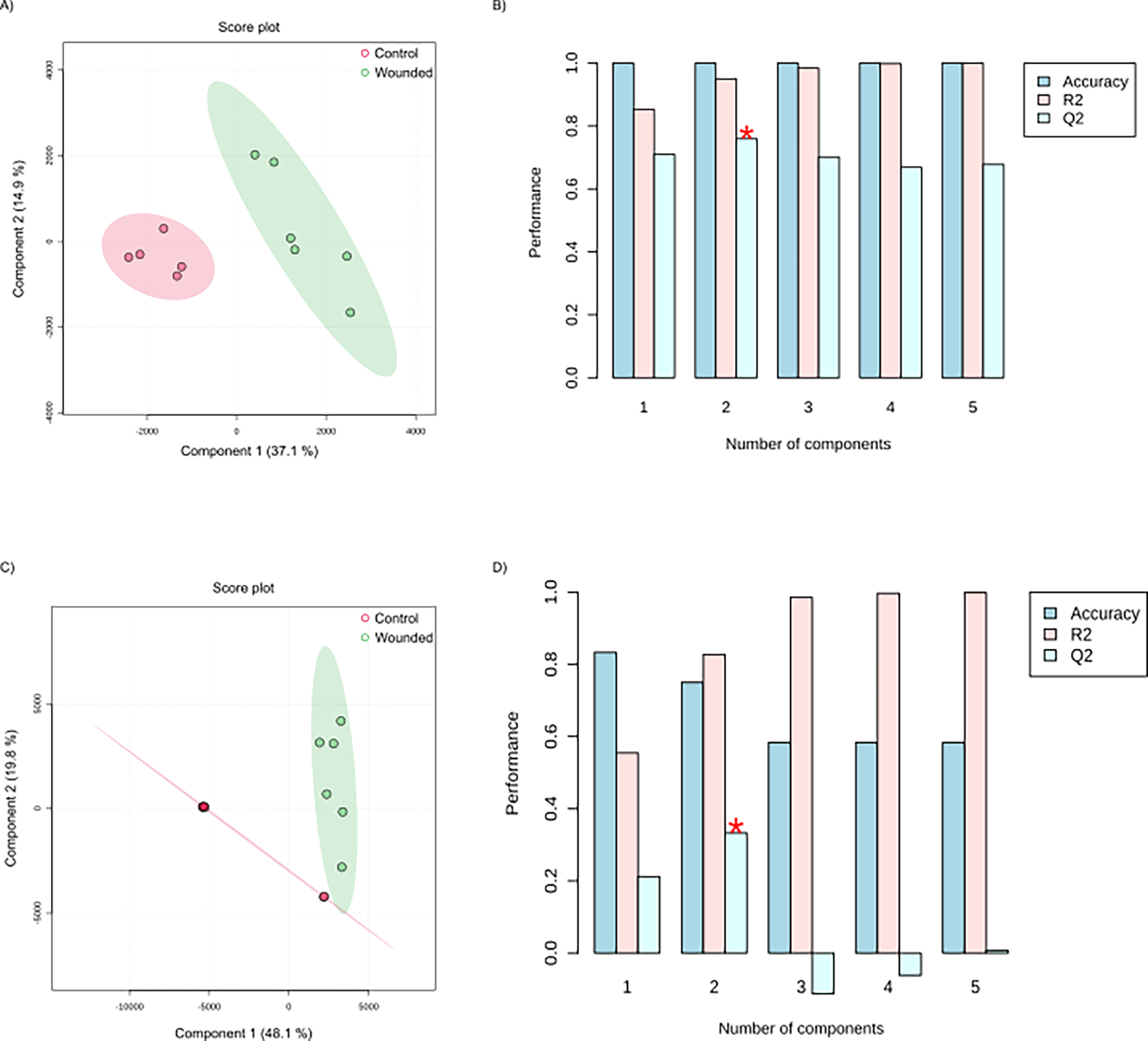

Supplement: Supplementary Figure 1 — (A) Multivariate analysis using supervised partial least squares discriminate analysis (PLS-DA) in separation of control and wounded leaves from broccoli leaves harvested at ZT0. (B) Cross validation analysis indicated that two components model was the optimal model for broccoli samples. (C) PLS-DA analysis of control and wounded leaves from turnip greens harvested at ZT8. (D) Cross validation analysis indicated no discrimination between turnip greens samples. Colored circles represent 95% confidence intervals. Colored dots represent individual samples. The component that best classifies the model is shown with red asterisk. [file Image_1.PNG]

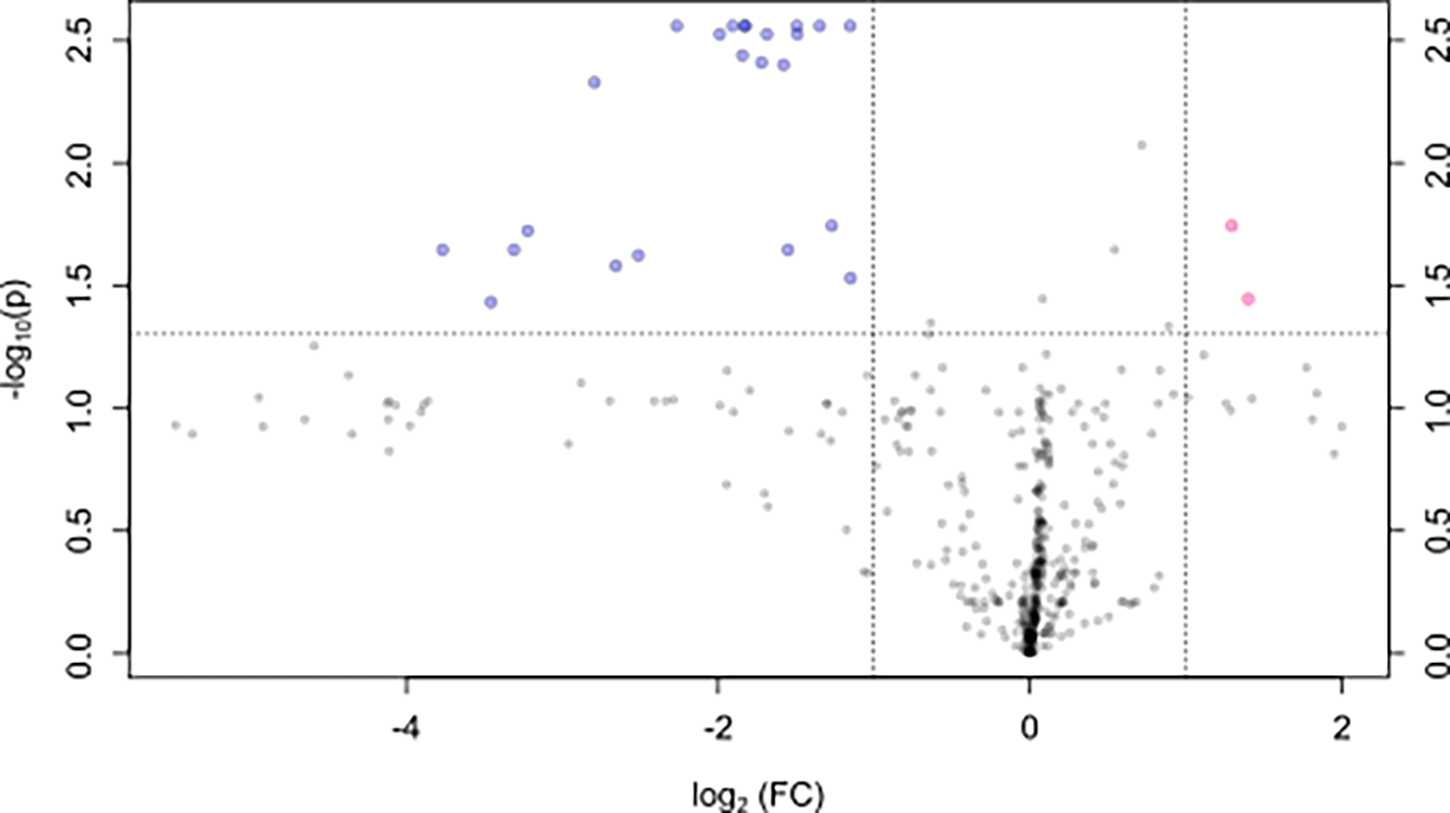

Supplement: Supplementary Figure 2 — Volcano plot displaying features that were most differentially expressed at wounded broccoli plants relative to control at ZT0. [file Image_2.PNG]
